# Controlling the Gate: The Functions of the Cytoskeleton in Stomatal Movement

**DOI:** 10.3389/fpls.2022.849729

**Published:** 2022-02-23

**Authors:** Yihao Li, Xin Zhang, Yi Zhang, Haiyun Ren

**Affiliations:** ^1^Center for Biological Science and Technology, Guangdong Zhuhai-Macao Joint Biotech Laboratory, Advanced Institute of Natural Science, Beijing Normal University, Zhuhai, China; ^2^Key Laboratory of Cell Proliferation and Regulation Biology of Ministry of Education, College of Life Sciences, Beijing Normal University, Beijing, China

**Keywords:** stomatal movement, actin filament, microtubule, actin-binding proteins, microtubule-associated proteins

## Abstract

Stomata are specialized epidermal structures composed of two guard cells and are involved in gas and water exchange between plants and the environment and pathogen entry into the plant interior. Stomatal movement is a response to many internal and external stimuli to increase adaptability to environmental change. The cytoskeleton, including actin filaments and microtubules, is highly dynamic in guard cells during stomatal movement, and the destruction of the cytoskeleton interferes with stomatal movement. In this review, we discuss recent progress on the organization and dynamics of actin filaments and microtubule network in guard cells, and we pay special attention to cytoskeletal-associated protein-mediated cytoskeletal rearrangements during stomatal movement. We also discuss the potential mechanisms of stomatal movement in relation to the cytoskeleton and attempt to provide a foundation for further research in this field.

## Introduction

The plant leaf epidermis and cuticle protect water against transpiration in relatively dry terrestrial environments but also limit gas exchange with the external environment for photosynthesis. Plants have evolved stomata on the leaf and stem epidermis; these structures consist of two kidney-shaped or dumbbell-shaped guard cells and are responsible for 95% gas exchange between the external atmosphere and the interior of the leaf (Keenan et al., 2013; Lawson and Matthews, 2020). Furthermore, stomata provide major sites for pathogen entry (Gudesblat et al., 2009; Zeng et al., 2010; Melotto et al., 2017). Plants are able to adjust stomatal opening and closure in response to environmental changes (Hetherington and Woodward, 2003; Murata et al., 2015). Hence, an attractive research system for investigations of signal transduction and physiological responses has been developed on the basis of stomatal functions.

The key factor driving stomatal movement is the turgor pressure change provoked by ions and water across plasma and vacuolar membranes, giving rise to swelling or deflation of the cells and opening or closing of the pores (Kollist et al., 2014; Woolfenden et al., 2018; Lawson and Matthews, 2020). In recent decades, a series of ion channels and transporters and their upstream regulators fine-tuning osmotic pressure in guard cells have been identified (Kollist et al., 2014; Lawson and Matthews, 2020). The activities of these channels and transporters depend on voltage sensing, ligand binding, or protein posttranslational modification. For example, the H^+^-ATPase AHA1 is activated by blue light, which leads to hyperpolarization of the plasma membrane in guard cells (Kinoshita and Shimazaki, 1999; Kinoshita et al., 2001; Hayashi et al., 2011). The change in membrane potential drives K^+^ influx through KAT1/2voltage-gated K^+^ channels, accompanied by anion Cl^−^ and malate influx (Lebaudy et al., 2010; Yamauchi et al., 2016). Increased levels of osmotically active substances further increase water uptake, resulting in the inflation of guard cells and stomatal opening.

Emerging studies provide evidences indicating that the cytoskeleton, including actin filaments (AFs) and microtubules (MTs), is considered as an important factor involved in stomatal movement, possibly *via* affecting turgor pressure in guard cells (Zhang and Fan, 2009; Khanna et al., 2014). The cytoskeleton participates in cell division and cell wall synthesis, which affect guard cell shape, structure, and mechanics (Galatis and Apostolakos, 2004; Panteris et al., 2018; Woolfenden et al., 2018; Muroyama et al., 2020). MTs guide cellulose synthesis complexes and determine cellulose microfibril orientation, which might provide high tensile strength in guard cells during stomatal movement (Woolfenden et al., 2018). On the other hand, the cytoskeleton undergoes rapid dynamic changes during stomatal movement, and stomatal movement is inhibited in cytoskeleton-deficient plants. In this review, we discuss current knowledge of cytoskeletal dynamics and their regulation in guard cells and aim to provide novel insights into the mechanisms of cytoskeleton-dependent stomatal movement.

## Dynamics and Functions of Actin Filaments in Guard Cells

Pharmacological inhibitors are commonly used to study the dynamics and functions of cytoskeleton and have demonstrated a prerequisite for actin remodeling in stomatal movement. Treatment with the AF stabilizers, such as phalloidin or jasplakinolide, inhibits stomatal closure induced by ABA, H_2_O_2_, and darkness, and phalloidin (but not jasplakinolide) also inhibited light-induced stomatal opening (Kim et al., 1995; MacRobbie and Kurup, 2007; Li et al., 2014). In contrast, the application of the AF-depolymerizing agent latrunculin B (but not cytochalasin B or D) accelerates ABA-induced stomatal closure, whereas cytochalasin B and D facilitate light-induced stomatal opening (Kim et al., 1995; MacRobbie and Kurup, 2007). The different effects of phalloidin versus jasplakinolide treatment and latrunculin B versus cytochalasin treatment may be due to the different mechanisms on AFs of these drugs and different drug sensitivity for plant materials. For example, latrunculin B binds to actin monomers and cytochalasin D binds to the barbed end of AF to inhibit AF polymerization. Nevertheless, these pharmacological experiments support the necessity of AFs in both stomatal opening and closing. The interconversion and configurations of AFs are highly correlated with the movement of stomatal aperture. Based on orientation, bundling and density, AF configurations in guard cells have been classified into three types during stomatal movement: (1) “radial arrays” or “radial bundles”: sparse AFs or bundles are distributed radially from stomatal pores in three-dimensional projection images, and actually cortical AFs are arranged in a circular pattern in cross-sections; (2) “random meshwork”: AFs are randomly distributed and organized into mesh-like networks with a high density; and (3) “longitudinal arrays”: most AFs form long bundles aligned in the longitudinal direction in guard cells (Gao et al., 2008; Higaki et al., 2010; Li et al., 2014; Shimono et al., 2016; Isner et al., 2017). In general, radial array and radial bundle configurations are more likely to be present in open stomata; filaments are reorganized into promiscuous mesh-like arrangements in the transition state; and longitudinal array configurations are dominant in closed stomata (Higaki et al., 2010; Shimono et al., 2016). Consistently, live-cell imaging of AF configurations in stomatal apertures revealed that AF remodeling during stomatal movement can be summarized as follows: the cortical radial AFs in open stomata first disassemble and are randomly distributed in response to environmental or endogenous signals, followed by reassembly into long bundles that are parallel to the long axis of guard cells, leading to stomatal closure. By observing AF behaviors in guard cells in the early stage of stomatal closure induced by the bacterial flagellin peptide flg22 at the single-filament level, Zou et al. observed that the AF-bundling frequency decreased while the severing frequency increased, which contributed to rapid AF disassembly (Zou et al., 2021). Cotreatment with phalloidin inhibits ABA or darkness-induced stomatal closure, supporting the notion that AF disassembly may be a critical step for initializing stomatal closure (Gao et al., 2008).

AF dynamics is important factor regulating the activity of ion channels and NADPH oxidase at the membrane that participates in stomata movement. A study in cytochalasin D-treated guard cells evaluated the activity of plasma membrane-localized osmo-sensitive voltage-dependent inward K^+^ channels and Ca^2+^-permeable channels at the single-channel level and found that the whole-cell current was increased (Hwang et al., 1997; Liu and Luan, 1998; Zhang and Fan, 2009). In contrast, AF stabilizer phalloidin treatment restrained inward whole-cell Ca^2+^ current (Zhang and Fan, 2009). Latrunculin B treatment enhances the vacuolar efflux transient induced by 10 μM ABA (Ca^2+^ influx rather than internal Ca^2+^ release at this concentration of ABA) but inhibits that induced by 0.1 μM ABA (triggering internal Ca^2+^ release rather than Ca^2+^ influx), indicating that AFs also regulate vacuolar ion efflux transient (MacRobbie and Kurup, 2007). The depolymerization of AFs by latrunculin B also enhances ABA-induced H_2_O_2_ production through increasing the activity of plasma membrane-localized NADPH oxidase RbohD (Li et al., 2014). Whereas it is still poorly understood how the activity of these proteins is influenced by AF turnover, several possible mechanisms could be tested. A tethering model has been proposed in mammalian cells and *Drosophila*, where the channel is gated by tethering to the cytoskeleton for mechanosensing (Jiang et al., 2021). For examples, AFs tether Piezo channels in mammalian cells and MTs tether NOMPC channels (belonging to the TRP family) in *Drosophila* for mechanogating (Zhang et al., 2015; Wang et al., 2020). Whether a similar mechanism whereby the cytoskeleton directly controls the activity of ion channels is conserved in guard cells still awaits further investigation. The distribution patterns and dynamic properties are also critical for membrane protein function. A recent study revealed that AFs and MTs participate in the dynamics of the aquaporin AtPIP2;1 at the plasma membrane during flg22-induced stomatal closure. Lat B treatment promotes the aggregation of AtPIP2;1 at the plasma membrane and accelerates water loss in response to flg22 (Cui et al., 2021). This study provides a new point of view on the activity of plasma membrane proteins regulated by the cytoskeleton.

Vacuoles play a critical role in the regulation of turgor pressure in guard cells. Large vacuoles invaginate to form transvascular strands in opened stomata and split into small vacuoles during stomatal closure, which contributes to changes in the volume of guard cells and the excessive storage of membrane materials (Gao et al., 2005; Tanaka et al., 2007; Yang et al., 2021). AF dynamics is also involved in regulating the morphology of vacuoles in guard cells. AFs colocalize with tonoplasts and encircle small vacuoles (Li et al., 2013). Both depolymerizing or stabilizing AFs by pharmacological agents inhibit the fusion of small vacuoles in guard cells during stomatal opening, as in other cell types (Higaki et al., 2006; Li et al., 2013; Scheuring et al., 2016). SCAB1 is a plant-specific actin-binding protein that can bind, stabilize, and cross-link AFs through dimerization (Zhao et al., 2011; Zhang et al., 2012b; Wang et al., 2015). The mutation of *SCAB1* affects the morphological remodeling of the vacuole, and an increased number of transvascular strands appear in the guard cells of *scab1* mutants (Yang et al., 2021). The ARP2/3 multi-subunit complex, containing two actin-related proteins ARP2 and ARP3, and five other actin-related protein complex units (ARPC1-5), is an important nucleation-promoting and branching factor for AFs (Deeks and Hussey, 2005; Yanagisawa et al., 2013). Vacuole fusion is impaired during stomatal opening in AF nucleator *arp2* and *arp3* mutants owing to abnormal reorganization of AFs (Li et al., 2013). AFs are also involved in vesicle trafficking from Golgi and release to vacuole (Kim et al., 2005; Akkerman et al., 2011) and the disassembly of AFs affects cargo trafficking from the Golgi complex to the vacuole (Kim et al., 2005). AP3M, the medium subunit of the AP3 complex, serves as an AF-severing protein that participates AF reorganization and vacuole morphology. The mutation of *AP3M* alters AF status in guard cells and abolishes the transportation of Golgi cargoes, such as the sucrose exporter SUC4, to the tonoplast, leading to defects in stomatal closure under drought stress (Zheng et al., 2019).

## Response of Actin-Binding Proteins to Upstream Signals in Guard Cells

Actin-binding proteins (ABPs), which modulate AF nucleation, severing, bundling, polymerization, and depolymerization, alter AF dynamics to markedly respond to environmental changes (Li et al., 2015). Recent studies have demonstrated that several ABPs are involved in the regulation of stomatal movement. Mutations in the ARP2/3 complex subunits *arpc4, arpc5, arpc2* (referred to as *hsr3*), *arp2* (referred to as *wrm*), or *arp3* (referred to as *dis1*) cause a similar phenotype: reduced or abolished dark-, ABA-, and H_2_O_2_-induced stomatal closure and retarded light-induced stomatal opening (Jiang et al., 2012; Li et al., 2013, 2014; Isner et al., 2017). *arpc4* and *arpc5* mutants show sparser but thicker actin bundles in the guard cells of both opened and closed stomata, suggesting that AFs tend to form bundles in the mutants. AF reorganization is also hysteretic during stomatal closure in the mutants (Li et al., 2014). The ABA-induced mesh-like network organization of AFs is suppressed and thus a more radial array of AFs is retained in the *arpc2* mutant compared to the wild type (Jiang et al., 2012). Cytochalasin D can restore the defect of stomatal closure in response to ABA in the *hsr3* mutant, suggesting that ABA-induced AF disassembly is disrupted in *hsr3* (Jiang et al., 2012). The ARP2/3 complex is in an intrinsically inactive conformation, which could be converted to an active conformation by the WAVE/SCAR (WASP family Verprolin homologous protein/Suppressor of cAMP Repressor) complex (Frank et al., 2004; Deeks and Hussey, 2005; Yanagisawa et al., 2013). A mutation in the *PIR1* gene encoding a subunit of the SCAR/WAVE complex results in reduced dark-induced stomatal closure, while a normal response to ABA or CaCl_2_ is retained, and the dark-insensitive phenotype can be restored by latrunculin B or cytochalasin D treatment (Isner et al., 2017). These results indicate that the ARP2/3 complex, along with its upstream regulator, the SCAR/WAVE complex, is required for stomatal movement through their roles in modulating AF disorganization and remodeling. However, it is still unclear how the ARP2/3 complex and the SCAR/WAVE complex contribute to AF disassembly or rearrangement in guard cells.

Actin-Depolymerizing Factor (ADF) family is a conserved class of ABPs that are involved in plant development and stress responses. The *Arabidopsis* genome encodes 11 ADF genes categorized into four subclasses (Inada, 2017; Nan et al., 2017), among which most members have conserved actin filament depolymerizing functions, while subclass III members have instead evolved filament bundling functions (Nan et al., 2017). ADF4, a member of subclass I, regulates stomatal closure in response to ABA. The *adf4* mutant displays lower AF occupancy but thicker bundles in guard cells than the wild type (Zhao et al., 2016). In contrast, the subclass III member ADF5 regulates drought- and ABA-induced stomatal closure *via* its AF-bundling activity. ABA and drought directly promote ADF5 expression mediated by ABF/AREB transcription factors in *Arabidopsis* and *Populus* (Qian et al., 2019; Yang et al., 2020). The guard cells of the *adf5* mutant exhibit fewer and thinner bundles of actin filaments in open stomata and delayed actin filament reorganization during stomatal closure (Qian et al., 2019). The activity of ADF proteins is governed by many factors, including pH, phosphorylation modifications, and phosphoinositide binding (Inada, 2017). The phosphorylation of the conserved sixth serine (Ser-6) of plant ADF1 and ADF4 inhibits their binding to AFs and therefore abolishes the AF-disassembling activity (Porter et al., 2012; Dong and Hong, 2013). Recently, Shi et al. reported that ABA accumulates and inhibits PP2Cs activity through the ABA-PYLs-PP2Cs complex, resulting in the activation of CKL2 in guard cells (Shi et al., 2021). The ABA-activated CKL2 (Casein Kinase 1-Like Protein 2) phosphorylates ADF4 at Ser-6, which contributes to AF reorganization in ABA- and drought-induced stomatal closure (Zhao et al., 2016). More AF-severing events can be observed in the *ckl2* mutant, and the severing activity of ADF4 is inhibited in the presence of CKL2 *in vitro* (Zhao et al., 2016). Ser-6, Ser-105, and Ser-106 of ADF4 can be phosphorylated by calcium-dependent protein kinase 3 (CPK3), which is required for the association with AFs, and stomatal immunity and pattern-triggered immunity (Lu et al., 2020). Moreover, the activity of ADFs is pH sensitive (Zhang et al., 2001; Nan et al., 2017; Wioland et al., 2019). As stomatal movement is associated with changes in intracellular pH due to the influx or efflux of proton, it is plausible to speculate that pH changes may control stomatal movement at least partially by modulating the activity of ADFs.

Villin belongs to a multifunctional villin/gelsolin/fragmin superfamily that exhibits multiple biochemical activities, including AF bundling, Ca^2+^-dependent AF severing, and barbed end capping (Huang et al., 2015). A recent study detailed investigated the functions of Villin3 in stomatal immunity. Zou et al. reported that the *vln3* mutant showed reduced AF turnover in guard cells treated with flg22, resulting in failure to close stomata upon bacterial infection (Zou et al., 2021). Flg22-activated MPK3/MPK6 phosphorylates Villin3 at Ser779 to specifically enhance its severing activity. Neither reduced AF bundling nor increased severing is observed in the guard cells of *vln3* or *mpk3/6* double mutant plants in the early stage of flg22 treatment compared to wild type. A phosphorylation mimic version of Villin3 can restore AF dynamics and stomatal movement in the *vln3* and *mpk3/6* mutants to the WT level, supporting the importance of VLN3 phosphorylation by MPK3/6 in modulating actin remodeling to activate stomatal defense in Arabidopsis (Zou et al., 2021).

Phosphoinositide exhibits an important function in stomatal movement and ABP activity regulation. The light-induced accumulation of PI (4,5)P2 triggers stomatal opening (Lee et al., 2007). Phosphatidylinositol 4-phosphate (PI4P), a precursor of PI(4,5)P2, and phosphatidylinositol 3-phosphate (PI3P) are required for light-induced stomatal opening and ABA-induced stomatal closure and modulate actin dynamics in guard cells (Jung et al., 2002; Choi et al., 2008). The PI3P and PI4P synthesis inhibitors LY294002 and wortmannin inhibit the ABA-induced random orientation of AF arrays in the guard cells of dayflower (*Commelina communis*) (Choi et al., 2008). Both overexpression and mutation of the AF cross-linking protein SCAB1 have similar effects, including a reduced rate of actin reorganization and a delay of stomatal closure induced by ABA. However, SCAB1 overexpression results in a higher frequency of bundled actin forms compared to the control, while the *scab1* mutant shows similar actin filament reorganization to the wild type (Zhao et al., 2011). Recent research has revealed that SCAB1 binds to PI3P through its RXLR-dEER PI3P-binding motifs. PI3P binding inhibits SCAB1 oligomerization, which further impairs AF destabilization and reorganization during ABA-induced stomatal closure (Yang et al., 2021). Several members of other ABP families, such as Villins and ADFs, as well as the upstream regulator of ARP2/3 complex, the WAVE/SCAR complex (Xiang et al., 2007; Zhao et al., 2010; Qin et al., 2021), bind to and are regulated by phospholipids. These interactions pose the possibility that phospholipids may regulate stomatal movement *via* multiple mechanism.

## Microtubule Organization Changes During Stomatal Movement

The function of MTs in stomatal movement has long been debated due to conflicting results from different experiments (Assmann and Baskin, 1998; Fukuda et al., 1998; Marcus et al., 2001). Assmann et al. reported that neither the microtubule-destabilizing drug colchicine nor the stabilization drug paclitaxel had any effect on stomatal opening or closing in epidermal peels of *Vicia faba* (Assmann and Baskin, 1998). Contrary results were observed by Fukuda et al. and Marcus et al., where they showed that the microtubule-destabilizing drugs propyzamide, oryzalin, and trifluralin inhibited stomatal opening and that paclitaxel treatment suppressed stomatal closing in the same material (Fukuda et al., 1998; Marcus et al., 2001). It is still unclear why these experiments resulted in totally distinct conclusion. Nevertheless, accumulating evidences from recent decades favor the notion that the MT arrays participate in stomatal movement. During light-induced stomatal opening, oryzalin treatment blocks stomatal opening (Eisinger et al., 2012a; Qu et al., 2017), while stabilization of microtubules by paclitaxel accelerates stomatal opening in a dose-dependent manner (Qu et al., 2017). ABA-, darkness-, and NO-induced stomatal closure was markedly inhibited by cotreatment with paclitaxel, but no significant changes were observed when oryzalin was applied (Zhang et al., 2008; Eisinger et al., 2012a; Qu et al., 2017; Biel et al., 2020). It has also been reported that treatment with oryzalin alone affects stomatal closure (Khanna et al., 2014).

Live-cell imaging revealed that the number and arrangement pattern of MTs in guard cells are correlated with the stomatal aperture. In open stomata, MTs radiate from the ventral side to the dorsal side in a more parallel, straighter and denser fashion relative to AF organization (Eisinger et al., 2012a; Qu et al., 2017; Biel et al., 2020). Following stomatal closing, MT structures decrease in number and become diffused. Some studies have shown that MTs completely depolymerized (Qu et al., 2017; Yu et al., 2020), while other studies have indicated that MTs are still present, but with reduced density, in closed stomata (Khanna et al., 2014; Biel et al., 2020; Yu et al., 2020; Dou et al., 2021). In the latter case, observable MTs tend to have a longitudinal arrangement and become crisscrossed or randomly patterned near the ventral side (Fukuda et al., 1998; Lahav et al., 2004; Zhang et al., 2008; Eisinger et al., 2012a; Biel et al., 2020). By using end-binding protein 1 (EB1) to label the growing plus ends of microtubules, it was observed that there were no significant changes in the number of growing ends or the growth velocity rate of microtubules during stomatal closure. This observation suggested that the reduction of microtubule density during stomatal closure was most likely resulted from microtubule disassembly (Eisinger et al., 2012a,b). Recently, increasing observations indicate that cortical microtubules are sensitive to tensile stress (Hamant et al., 2008, 2019; Gorelova et al., 2021). Based on the observations from atomic force microscopy and finite element method simulations, MT organization is found to be consistent with the tensile pattern of guard cell (Sampathkumar et al., 2014; Gorelova et al., 2021). Thus, changes in microtubule organization may be a consequence of stomatal movement.

The functions of MTs in stomata movement are still poorly understood. MTs play essential roles in determining the arrangement of cellulose microfibrils and other non-cellulosic compounds in the cell walls, which provides mechanical properties for stomatal movement (Oda, 2015; Rui and Anderson, 2016; Yi et al., 2018). MT guides the trajectories of the cellulose synthesis complexes (CSCs) for cellulose synthesis at the cell surface (Gutierrez et al., 2009). Colocalization between CSCs and MTs is reduced and CSC motility speed increase during dark-induced stomata closure (Rui and Anderson, 2016). During stomatal movement, cellulose in guard cell walls undergoes reorganization from a more diffuse distribution in opened stomata to extensive bundles in the closed state (Rui and Anderson, 2016). MTs also determine the alignment mode of callose deposition and disassembly of MTs by oryzalin disturbs the pattern of callose deposition in the guard cell (Apostolakos et al., 2009). Thus, MT organization may impact on cell wall organization during stomatal movement and the detail mechanism deserves further investigation.

## Microtubule-Associated Proteins and Upstream Signaling in Regulating Microtubule Organization in Guard Cells

Several microtubule-associated proteins (MAPs) and their upstream regulators play vital roles in regulating MT organization and stomatal movement. WDL7, a member of the WAVE-DAMPENED2 (WVD2)/WVD2-LIKE family, directly binds to and bundles MTs *in vitro*. WDL7-overexpressing plants show delayed stomatal closure in response to ABA compared to WT plants. MTs are less sensitive to oryzalin- and ABA-induced MT disruption in WDL7-overexpressing guard cells, indicating that WDL7 serves as a MT stabilizer. Consistently, the *wdl7* mutant shows impairment of MT assembly and the stomatal opening response to light (Dou et al., 2021). WDL7 protein stability is regulated by ubiquitination. MREL57 (MICROTUBULE RELATED E3 LIGASE 57) directly targets and ubiquitinates WDL7 for degradation. *mrel57* mutant exhibits ABA insensitivity of stomatal closure and microtubule disassembly in guard cells (Dou et al., 2021). Several other ubiquitin E3 ligases, including JUL1 (JAV1-ASSOCIATED UBIQUITIN LIGASE1) and COP1 (CONSTITUTIVE PHOTOMORPHOGENIC 1), also participate in MT remodeling during stomatal movement (Khanna et al., 2014). JUL1 mediates ABA-induced microtubule disorganization and stomatal closure downstream of H_2_O_2_ and calcium. JUL1 binds to polymerized microtubules but not tubulin heterodimers (Yu et al., 2020). Darkness- and ABA-induced stomatal closure and MT disassembly are suppressed in the *cop1* mutant, and oryzalin is able to reduce this effect, indicating that the function of COP1 is critical for MT destabilization upon darkness and ABA treatment in guard cells (Mao et al., 2005; Khanna et al., 2014; Chen et al., 2021). It has been reported that COP1 directly ubiquitinates the MT stabilizer WDL3 in hypocotyl cells grown under darkness (Lian et al., 2017). Whether an analogous mechanism exists in guard cells requires further evaluation.

Phosphatidic acid (PA) has emerged as a vital signaling molecule involved in regulating cytoskeletal organization and dynamics under abiotic and biotic stresses, including the regulation of stomatal movement (Pleskot et al., 2013). PA is produced through the hydrolysis of phosphatidylcholine (PC) by phospholipase D (PLD) or the phosphorylation of diacylglycerol (DAG) by DAG kinase (Testerink and Munnik, 2011; Pleskot et al., 2013). Heat shock triggers ROS production to stimulate the activity of plasma membrane-localized PLDδ, and PLDδ directly binds to and disassembles MTs and causes stomatal closure under heat stress (Zhang et al., 2017; Song et al., 2020). The mutation of *PLDα1* maintains stomatal opening and well-organized MTs in the presence of ABA. Exogenous application of PA but not PC, PE or PS promotes microtubule depolymerization in stomatal cells, suggesting that PA may regulate stomatal movement through its impact on MTs (Jiang et al., 2014). However, the molecular mechanism underlying how PA induces MT depolymerization remains unclear. It has been reported that PA binds to MAP65-1 and promotes MT polymerization and bundling under salt stress (Zhang et al., 2012a). It still needs to be explored whether PA activates a MAP or a signaling pathway to disassemble MTs in guard cells.

SINE1 and SINE2 (SUN-INTERACTING NUCLEAR ENVELOPE PROTEIN 1 AND2), which are two components of the plant LINC (LINKER OF NUCLEOSKELETON AND CYTOSKELETON) complex, are involved in regulating the reorganization of MTs in guard cells during stomatal movement. The loss of function of either *SINE1* or *SINE2* results in a disordered MT organization in open stomata (Biel et al., 2020). There are fewer MT filaments or bundles in *sine 1* or *sine 2* mutants compared to the wild type during stomatal closure, leading to insensitivity to ABA-induced stomatal closure (Biel et al., 2020). Translationally controlled tumor protein (TCTP) is a calcium- and tubulin-binding protein, and the binding of calcium facilitates TCTP binding to microtubules. The overexpression of TCTP increases ABA- and calcium-induced stomatal closure ratios to limit water evaporation by accelerating MT depolymerization (Kim et al., 2012). The detailed characterization of the biochemical activities of SINE1/2 and TCTP toward MTs should be further defined.

## Conclusion and Perspective

**Figure 1:**
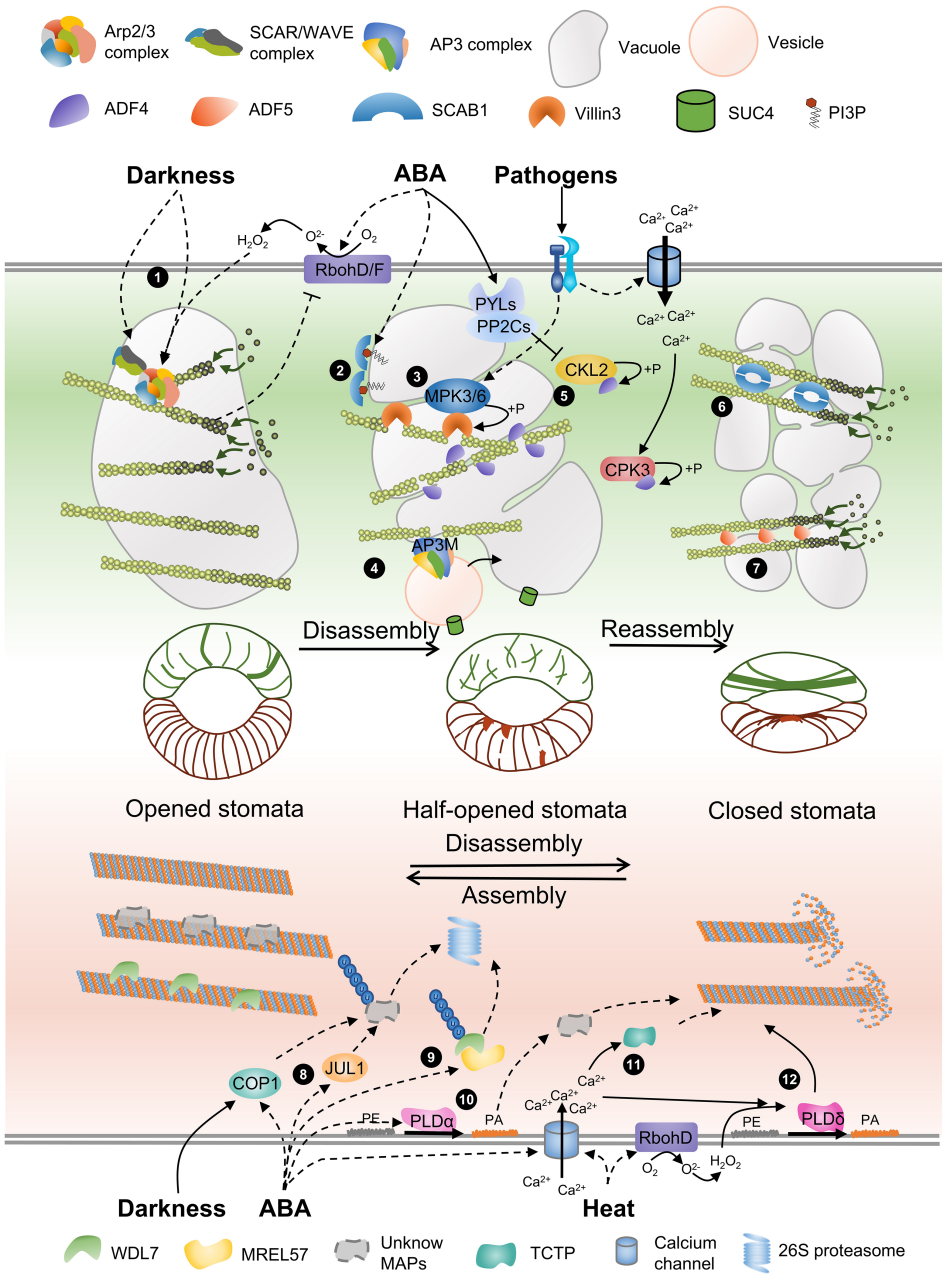
A schematic model of AF and MT remodeling accompanied by vacuole morphology during stomatal closure. The schematic model of stomata in the middle of the panel shows the distribution of AFs and MTs in guard cells with different stomatal apertures. The gray circular charts present the different morphology of vacuole during stomata movement. Several actin-binding proteins and microtubule-associated proteins are involved in regulating cytoskeletal rearrangement. (1) The Arp2/3 complex and the activator SCAR/WAVE complex are required for AF nucleation and branching, and darkness-induced stomata closure. ABA-triggered H_2_O_2_ generation by RbohD/RbohF regulates AF dynamics through the Arp2/3 complex but does not regulate the SCAR/WAVE complex, and AF feedback regulates H_2_O_2_ production. (2) ABA-triggered PI3P biosynthesis inhibits the oligomerization of SCAB1. (3) A pathogen triggers MPK3/MPK6 activation, and Villin3 is then phosphorylated to increase AF-severing activity. (4) AP3M of the AP3 complex severs AFs and regulates vesicles from Golgi carrying SUC4 fused to the tonoplast. (5) ADF4 binds to and severs AFs, and ABA-activated CKL2 and calcium-activated CPK32 induced by pathogens phosphorylate ADF4 to inhibit its activity and promote AF reorganization. (6 and 7) SCAB1 dimers and ADF5 monomers bundle and stabilize AFs and promote AF reassembly. (8) JUL1 and COP1, two other E3 ubiquitin ligases, may control the degradation of unknown MT-stabilizing factors and promote MT disassembly. (9) WDL7 stabilizes MTs in open stomata. The E3 ubiquitin ligase MREL57 interacts with and ubiquitinates WDL7 for 26S proteasome degradation during ABA-induced stomatal closure. (10) ABA-triggered PA produced by PLDα induces MT depolymerization through an unknown mechanism. (11) An ABA-induced increase in cytosolic calcium increases TCTP binding to MTs and MT destabilization. (12) Heat shock stimulates H_2_O_2_ production, and calcium influx activates PLDδ, which is required for MT depolymerization.

Based on the knowledge available, we propose a model of current progress about AF and MT dynamics that are regulated by different functional ABPs and MAPs during stomatal movement. In opened stomata, the ARP2/3 complex is activated by WAVE/SCAR complex and promotes AF nucleation and branching, contributing to AF network formation. In the transition stage of stomatal closure, AF-severing factors, such ADF4 and Villin3, lead to AF depolymerization, and an increasing content of PI3P inhibits the cross-linking function of SCAB1, promoting the disassembly of the AF network. Subsequently, the phosphorylation of ADF4 inhibits its severing activity, and the upregulation of *ADF5* and SCAB1 dimer contributes to the formation of long AF bundles in closed stomata. WDL7 binds and stabiles MT in opened stomata. During ABA-induced stomatal closing, E3 ligase MREL57 ubiquitinates WDL7 for degradation; ABA-triggered calcium influx activated TCTP to stimulate MT disassembly. PLDδ activated in an H_2_O_2_- and calcium-dependent manner and disassembles MTs upon heat stress (Figure 1). Although the dynamic distribution of AFs and MTs during stomatal movement has been reported, the underlying molecular mechanism is still not well understood. Additional ABPs, MAPs and upstream proteins involved in stomatal movement need to be detailed analyzed, which will facilitate to dissect the roles of cytoskeleton in transducing environmental signals to stomatal movement. The arrangement mode of AFs and MTs is somewhat similar in guard cells, posing the possibility that they may coordinate to control stomata movement. Some ABPs, such as formin proteins, have been reported to interact with both AFs and MTs (Li et al., 2010; Wang et al., 2013; Sun et al., 2017). It is worthy to further investigate the interaction between AFs and MTs, as well as the underlying molecular mechanism in stomata. Although many evidences have demonstrated the potential for cytoskeleton in regulating the activity of ion channels, vesicles trafficking, and cell wall dynamics of guard cell, the detailed mechanisms need to be explored in further for advancing our understanding of the cytoskeleton function contributed to stomatal movement.

Stomata are of great importance in the response and adaptation of plants to environmental changes. Previous research in this context has mainly focused on kidney-shaped guard cells in *Arabidopsis*, *Vicia*, and tobacco. The distribution of AFs and MTs in dumbbell-shaped guard cells in most crop plants is different from that in kidney-shaped guard cells (Spiegelhalder and Raissig, 2021). Advances in research on cytoskeletal dynamics in dumbbell-shaped guard cells remain relatively stagnant. Studies on the mechanisms of stomatal movement in crops will help to improve the efficiency of carbon assimilation and water use under the trend of global warming.

## Author Contributions

YL and XZ collected the references and wrote the manuscript. YZ and HR revised the manuscript. All authors have read and approved the final manuscript.

## Funding

This work was supported by the National Natural Science Foundation of China (91854206 and 32170335 to HR; 31870174 and 32070194 to YZ).

## Conflict of Interest

The authors declare that the research was conducted in the absence of any commercial or financial relationships that could be construed as a potential conflict of interest.

## Publisher’s Note

All claims expressed in this article are solely those of the authors and do not necessarily represent those of their affiliated organizations, or those of the publisher, the editors and the reviewers. Any product that may be evaluated in this article, or claim that may be made by its manufacturer, is not guaranteed or endorsed by the publisher.
